# Decision tree model for predicting in‐hospital cardiac arrest among patients admitted with acute coronary syndrome

**DOI:** 10.1002/clc.23255

**Published:** 2019-09-11

**Authors:** Hong Li, Ting Ting Wu, Dong Liang Yang, Yang Song Guo, Pei Chang Liu, Yuan Chen, Li Ping Xiao

**Affiliations:** ^1^ Department of Nursing Fujian Provincial Hospital Fujian China; ^2^ Department of Nursing Fujian Health College Fujian China; ^3^ Department of General Education Courses Cangzhou Medical College Hebei China; ^4^ Department of Cardiovascular Medicine Fujian Provincial Hospital Fujian China; ^5^ Department of Anesthesiology Union Hospital Affiliated to Fujian Medical University Fujian China; ^6^ Department of Nursing Xiamen Cardiovascular Disease Hospital Xiamen China; ^7^ Department of Nursing First Hospital of Longyan Longyan China

**Keywords:** acute coronary syndrome, decision tree model, in‐hospital cardiac arrest

## Abstract

**Background:**

In‐hospital cardiac arrest (IHCA) may be preventable, with patients often showing signs of physiological deterioration before an event. Our objective was to develop and validate a simple clinical prediction model to identify the IHCA risk among cardiac arrest (CA) patients hospitalized with acute coronary syndrome (ACS).

**Hypothesis:**

A predicting model could help to identify the risk of IHCA among patients admitted with ACS.

**Methods:**

We conducted a case‐control study and analyzed 21 337 adult ACS patients, of whom 164 had experienced CA. Vital signs, demographic, and laboratory data were extracted from the electronic health record. Decision tree analysis was applied with 10‐fold cross‐validation to predict the risk of IHCA.

**Results:**

The decision tree analysis detected seven explanatory variables, and the variables' importance is as follows: VitalPAC Early Warning Score (ViEWS), fatal arrhythmia, Killip class, cardiac troponin I, blood urea nitrogen, age, and diabetes. The development decision tree model demonstrated a sensitivity of 0.762, a specificity of 0.882, and an area under the receiver operating characteristic curve (AUC) of 0.844 (95% CI, 0.805 to 0.849). A 10‐fold cross‐validated risk estimate was 0.198, while the optimism‐corrected AUC was 0.823 (95% CI, 0.786 to 0.860).

**Conclusions:**

We have developed and internally validated a good discrimination decision tree model to predict the risk of IHCA. This simple prediction model may provide healthcare workers with a practical bedside tool and could positively impact decision‐making with regard to deteriorating patients with ACS.

## 1 INTRODUCTION

In‐hospital cardiac arrest (IHCA) is an infrequent, but life‐threatening complication of acute coronary syndrome (ACS). If healthcare staff do not recognize physical deterioration in patients, these patients could eventually go on to suffer cardiac arrest (CA), which could significantly enhance mortality, in comparison to non‐CA patients.^1^ Previous studies have demonstrated clinical deterioration hours before an adverse event actually occurs, therefore, healthcare staff should focus on detecting clinical deterioriation, thus preventing more adverse events.^2^, ^3^ A prediction model focused on the need for early risk stratification would obviously reduce the incidence of adverse clinical outcomes.

Risk factors in CA are associated with a multitude of variables, including age, heart rate, blood pressure, laboratory data, ST‐T abnormalities, heart rate variability, and Killip class.^4^, ^5^, ^6^ These risk factors could possibly help in guiding decision‐making and risk assessment for individual patients, but remain controversial.

Recently, there has been a movement in several countries to create a single unified risk score to assess hospitalized patients suffering CA as a result of a wide range of diseases.^7^, ^8^ However, it has demonstrated limited accuracy, leading to missed opportunities to identify the patients most likely to suffer CA, along with inefficient resources utilization, as it is not evidence‐based.

A decision‐tree model, which can be useful in developing a clinical prediction model, does not require assumptions about the underlying model and has excellent face validity for both clinicians and patients.^9^, ^10^ However, there is a paucity of decision tree analysis for predicting CA. The aim of this study was to develop an easy‐to‐use clinical prediction model that may help healthcare workers assess the risk of IHCA in patients admitted with ACS. For this purpose, we identified risk factors of CA that were present prior to an event, and developed and validated a prediction model for IHCA patients hospitalized with ACS.

## 2 METHODS

### 2.1 Study design and setting

We conducted a case‐cohort study review on all adult ACS patients discharged from January 2012 to December 2016 from three tertiary hospitals in Fujian province, China. These participant hospitals included two comprehensive hospitals and one specialist hospital, with approximately 1200, 2500, and 1900 annual admissions of ACS patients, respectively. All physicians and nurses were required to receive Advance Cardiac Life Support training to ensure their ability to resuscitate patients.

### 2.2 Study populations

At the participating hospitals, we identified a total of 21 337 ACS patients who had undergone a resuscitation attempt (chest compression and/or defibrillation) complicated by CA, from January 2012 to December 2016. In the case group, the inclusion criteria were: patients aged 18 years or older who had experienced a CA and had been admitted to hospital at least 24 hours later. Patients who were diagnosed with unstable angina (UA), acute ST‐segment elevation myocardial infarction (STEMI) and acute non‐ST‐segment elevation myocardial infarction (NSTEMI) were included. CA was defined by unresponsiveness, apnea, and the absence of a central palpable pulse due to pulseless ventricular tachycardia or ventricular fibrillation, pulseless electrical activity (PEA), or asystole. In the case group, the exclusion criteria were: patients with CA whose family caregivers had refused resuscitation, patients with missing data, or patients with prior out‐of‐hospital CA and who were transported to hospital with ongoing resuscitation, or CA that had occurred during an operation, or patients whose situation was complicated due to multiple organ failure and who were in the terminal stage of their disease. For patients with more than one IHCA during the same hospitalization, only the first arrest was included.

All ACS patients residing at a participant hospital who had not had a previous CA were eligible for inclusion in the control group. Patients were excluded if they had been discharged “against advice” or had missing data. Using a random number generator, we randomly selected the control subjects in a 1:3 ratio, with an ACS diagnosis, hospitalized in the same year and in the same hospital department (on the ward or in the ICU) as the CA patients in the case group.

### 2.3 Data collection

Our study group of seven, who collected data from April 2015 to January 2017, consisted of a cardiologist, anesthetist, nurse, nursing master's student, epidemiology master's student, and two nursing interns. The information was retrieved from patients' electronic medical records.

All data that were routinely collected included: Demographics (age, gender, height, weight, body mass index, smoking, length of day, others). Comorbidities (diagnosis of ACS, culprit artery, hypertension, diabetes, prior percutaneous transluminal coronary intervention, Charlson Comorbidity Index [CCI], Killip class, others). Vital signs (respiratory rate, blood pressure, heart rate, oxygen saturation, use of supplemental oxygen, temperature, and mental status in the prior 24 hours to CA. We calculated ViEWS using patients' recorded physiological parameters. ViEWS, developed in 2010 by Prytherch, is based on peripheral oxygen saturation and the presence of inhaled oxygen parameters, in addition to systolic blood pressure, pulse rate, respiratory rate, body temperature, and AVPU score. The scores vary from 0 to 3 for each parameter, and from 0 to 21 in total value.^11^ In our previous study, we reported that ViEWS was the most accurate tool for predicting CA, in comparison with the Modified Early Warning Score (MEWS) and National Early Warning Score (NEWS). 12 Laboratory values (white cell count, hemoglobin, platelet count, red cell count, lactate, sodium, potassium, chloride, bicarbonate, anion gap, blood urea nitrogen, glucose, serum creatinine, creatine kinase, creatine kinase‐MB, brain natriuretic peptide, cardiac troponin I, others). Imagological and electrocardiogram examinations (left ventricular ejection fraction, QTc interval, QRS durations, others).

### 2.4 Statistical analysis

Descriptive statistics were reported as mean ± SD, or median [interquartile range (IQR)] for continuous variables. For categorical variables, the percentages of patients in each category were calculated. Comparisons between categorical data were done by *χ* ^2^ test, and comparisons between continuous variables were done by Student's *t* test. As an estimate of effect size and variability, we have reported the odds ratio (OR) with a 95% confidence interval (CI).

We performed recursive portioning analysis to develop a decision tree model for IHCA prediction, which was used to separate patients into different homogeneous risk groups and to determine predictors for CA.^13^ We assessed overall model discrimination by using the area under the receiver operating characteristic curve (ROC). To cope with the overfitting and instability inherent in the decision tree, a 10‐fold cross‐validation procedure was applied. Missing values were treated with imputation by random forest. Statistical analyses were conducted using R software, version 3.4.3 (Chicago, Illinois). All significance tests used a two‐sided *P* value < 0.05.

## 3 RESULTS

### 3.1 Cohort description

**Figure 1:**
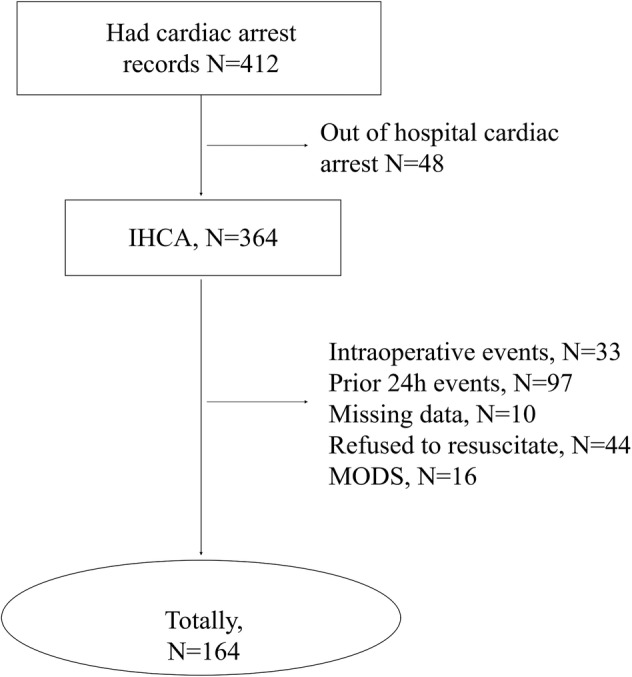
Flow chart of study participants. MODS, multiple organ dysfunction syndrome

Among the 21 337 ACS patient admissions combined within the four‐year study period, 412 ACS patients experienced an occurrence of IHCA in the participating hospitals. We excluded 44 cases in which family caregivers had refused resuscitation before the event; while in 48 cases, patients had an out‐of‐hospital CA before admission; 33 cases occurred during an operation; 16 cases occurred in the terminal stage of the disease; 24 cases occurred within 24 h of admission; and 10 cases had missing data. Our final population at the three hospitals was composed of 164 CA ACS patients in the case group, and 492 patients who had not suffered a CA, who were placed in the control group, as shown in Figure 1.

### 3.2 Baseline characteristics between the two groups

The baseline characteristics analysis between the two groups is shown in Table 1. There were no significant differences in hospital setting, hospital department, gender, diagnosis of ACS, hypertension, smoking, etc. (*P >* .05), however, age, diabetes, CCI and culprit artery were significantly different in the two groups (*P <* .05). According to previous reports, age,^5^, ^14^ diabetes,^15^, ^16^ and CCI^17^, ^18^were risk factors in predicting CA, so these may also be candidate predictors in our further analysis. The culprit artery of ACS cannot be clearly defined until an angiocardiography is done, which takes time and is unsuitable for early prediction, and so was not included in our analysis.

**Table 1:** Baseline characteristics of study patients

Variables	Case group (N [%])	Control group (N [%])	*Z/χ* ^*2*^	*P*
**Setting**	164	492		
A	90 (54.9)	266 (54.1)		.860
B	33 (20.1)	93 (18.9)	0.302	
C	41 (25.0)	133(27.0)		
**Department**				
ICU	103 (62.8)	297 (60.4)	0.308	.579
General ward	61 (37.2)	195 (39.6)		
**Age**	71.31 ± 11.51	65.67 ± 13.00	−5.368	<.001
**Gender**				
Male	120 (73.2)	385 (78.3)	1.517	.218
Female	44 (26.8)	107 (21.7)		
**Diagnosis of ACS**				
STEMI	72 (44.2)	206 (41.9)		.865
NSTEMI	77 (47.2)	240 (48.8)	0.290	
UA	14 (8.6)	46 (9.3)		
**Culprit artery**				
One‐vessel	41 (25.0)	158 (32.1)		.010
Two‐vessel	33 (20.1)	127 (25.8)	13.383	
Three‐vessel	47 (28.7)	135 (27.4)		
Left main coronary artery + multivessel	11 (6.7)	21 (4.3)		
Noncoronary angiography	32 (19.5)	51 (10.4)		
**Hypertension**				
Yes	107 (65.2)	303 (61.6)	0.702	.402
No	57 (34.8)	189 (38.4)		
**Hyperlipidemia**				
Yes	37 (22.6)	143 (29.1)	2.613	.129
No	127 (77.4)	349 (70.9)		
**Diabetes**				
Yes	67 (40.9)	137 (27.8)	9.713	.002
No	97 (59.1)	355 (72.2)		
**CCI**	3.27 ± 1.95	2.28 ± 1.57	−5.914	<.001
**Smoking**				
Yes	61 (37.2)	254 (51.6)	10.262	.001
No	103 (62.8)	238 (48.4)		
**Drinking**				
Yes	16 (9.8)	75 (15.3)	2.206	.106
No	147 (90.2)	416 (84.7)		

### 3.3 The decision tree model for predicting IHCA

**Figure 2:**
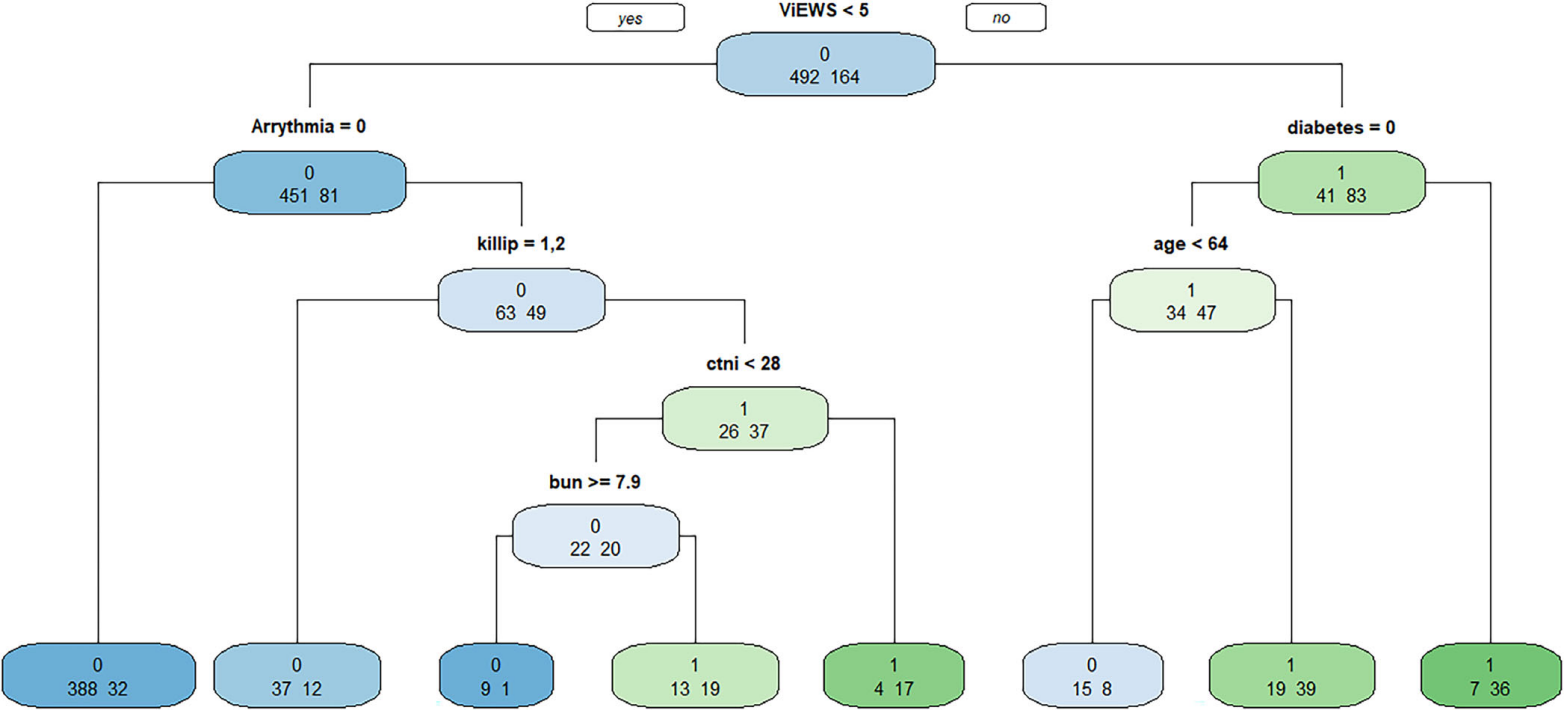
Decision tree model for predicting IHCA in patients admitted with ACS. Fatal arrhythmia: atrial arrhythmia = 1, borderline arrhythmia = 2, ventricular arrhythmia = 3; Killip class І = 1, II = 2, III = 3, IV = 4; Diabetes: yes = 1, no = 0. BUN, blood urea nitrogen; cTnI, cardiac troponin I

Figure 2 depicts the final decision‐tree model of the recursive partitioning analysis for predicting CA among patients admitted with ACS, ultimately generated into five layers and eight nodes. The analysis identified ViEWS as the most important discriminating factor, followed by fatal arrhythmia, Killip class, blood urea nitrogen, cardiac troponin I, age, and diabetes. These branch points permitted stratification into three prediction groups: high, intermediate, and low risk for CA prediction, as shown in Table 2.

**Table 2:** The risk group of decision tree model in predicting IHCA

Risk groups	Variables
High (70%‐100%)	
	ViEWS < 5, fatal arrhythmia, Killip > II, cTnI ≥ 28
	ViEWS ≥ 5, diabetes
Moderate (40%‐69%)	
	ViEWS < 5, fatal arrhythmia, Killip > II, cTnI < 28, BUN < 7.9
	ViEWS ≥ 5, no diabetes, age ≥ 64
Low (<40%)	
	ViEWS < 5, no fatal arrhythmia
	ViEWS < 5, fatal arrhythmia, Killip ≤ II
	ViEWS < 5, fatal arrhythmia, Killip > II, cTnI < 28, BUN ≥ 7.9
	ViEWS ≥ 5, no diabetes, age < 64

### 3.4 The discrimination of the development and internal validation model

**Figure 3:**
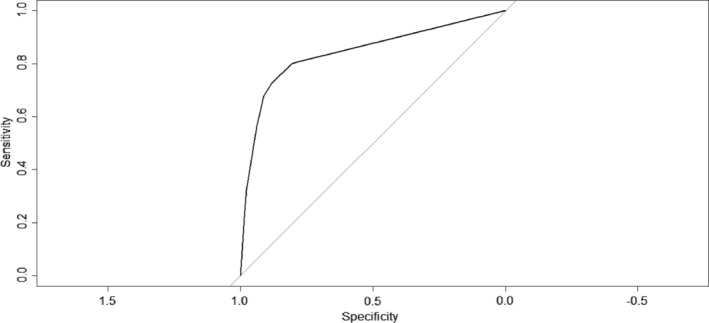
ROC of the development decision tree model. The receiver operating characteristic curve (AUC) of the development decision tree model is 0.844

We used ROC to evaluate the discrimination of the IHCA prediction model. The AUC for the decision tree model was 0.844 (95% CI, 0.805 to 0.849), shown in Figure 3, while the sensitivity and specificity were 0.762 and 0.882, respectively. The 10‐fold cross‐validated risk estimate was 0.198, the optimism‐corrected value of the area under the ROC was 0.823 (95% CI, 0.786 to 0.860).

## 4 DISCUSSION

CA is a rare, yet not negligible complication following hospitalization for ACS. For patients admitted with ACS, this study shows that the risk of IHCA can be reliably estimated by using a decision tree model consisting of seven clinical findings (ViEWS, fatal arrhythmia, Killip class, cardiac troponin I, blood urea nitrogen, age, diabetes) and could be implemented in the electronic health record to detect critically ill patients in real time. Discrimination of CA was good using a decision tree model and 10‐fold cross‐validation. This prediction model of a decision tree for ACS before IHCA could provide medical staff with a practical bedside tool for making medical decisions.

The most crucial predictor for CA was ViEWS. The National Institute for Health and Clinical Excellence recommended that physiologic tracking and trigger systems should be used to monitor all adult patients in an acute hospital setting^19^; most such systems are also known as early warning scores. ViEWS is an early warning score established by Prytherch et al.^11^ It was the best performing early warning score in a recent study that compared it to 33 other systems. Our previous study also proved that ViEWS is better at discriminating the risk of CA compared to two other common early warning scores.^12^

The second crucial predictor we evaluated was fatal arrhythmia. Studies have shown that among 45% to 55% of patients with inferior acute myocardial infarction, 8% to 35% of these patients have varying degrees of atrioventricular block.^20^ Atrioventricular block in patients with ACS is a common and serious complication. Compared to patients with non‐atrioventricular block, patients with atrioventricular block deteriorate quickly and are more likely to experience CA. Cao et al^21^ demonstrated that more than 6% of ACS patients had frequently occurring ventricular premature beats or ventricular tachycardia in the 48 hours prior to clinical deterioration. Although the use of PCI, coronary artery bypass grafting, and implanted automatic defibrillator has greatly reduced severe adverse events, ventricular arrhythmias remain a major risk factor for sudden CA in ACS patients. Bansilal et al^22^ and Liakopoulos et al^23^ reported that left bundle branch block is a major risk factor for both long‐term and short‐term prognosis of cardiovascular disease. Alkindi et al^24^ conducted a 23‐year follow‐up, from 1991 to 2013, of 768 patients with left bundle branch block. Their results show that left bundle branch block was an independent risk factor for in‐hospital death, with an OR of 2.96.

Including laboratory values in the decision tree model contributes important knowledge to the field, as most previous research has mainly focused on vital signs. We found that cardiac troponin I and blood urea nitrogen were significant predictors in the final model. Blood urea nitrogen is also a factor in other published criteria, such as electronic Cardiac Arrest Risk Triage (eCART)^5^ and the Acute Physiology and Chronic Health Evaluation II score (APCHEII).^25^ Matthew et al^5^ developed and validated eCART to predict CA or ICU transfer in ward. Their final model included blood urea nitrogen, anion gap, potassium, hemoglobin, white cell count, and platelet count. With the exception of blood urea nitrogen, the laboratory values have been quite inconsistent with our results, mainly due to the differences in participants. Matthew et al selected a wide range of patients hospitalized with different disease types, however, we included only ACS patients. Previous studies have demonstrated that cardiac troponin I elevations are associated with a higher number of cardiac events.^26^

Our decision tree model, which includes only seven variables and stratifies three groups, is a very simple and easily accessible model that allows any healthcare worker to predict IHCA patients hospitalized with ACS, with potential implications for identifying deteriorating patients. It is based on variables that include ViEWS, fatal arrhythmia, Killip class, cardiac troponin I, blood urea nitrogen, age and diabetes, which are easy to obtain from patient characteristics, ECG monitoring and laboratory tests; would not be limited by hospital conditions; and would be conducive to clinical practice. Our results can be distinguished from other models established by Matthew et al^5^ and Jonas et al,^4^ which use the algorithm of logistic regression. Decision tree model was given to the interest in generating a classification that would reasonably imitate authentic decision‐making. This means that, compared to a binary logistic regression, which postulates the existence of additive effects that contribute to explaining an outcome, decision trees factor in the existence of strong interactions between variables, and are better suited to elaborating decision‐making algorithms that follow the same structure.^27^

This study has certain limitations that must be taken into account. First, it is a fundamental case‐control study of medical history data, with the intrinsic limitations in precision this entails. Nevertheless, most of the events contemplated are solid and are faithfully recorded in the histories. Second, although the decision tree model contains only seven factors, ViEWS is a composite factor, including vital signs and consciousness. It will increase workload, so establishing an electronic physiological surveillance system may be more efficient. Third, a decision tree model can be weak, with unstable predictors in the case of a shortage of participants, so a larger sample size will be needed in a further study. Finally, some previously reported factors, such as imagological variables, biomarkers, electrocardiogram features (eg, lactate, right ventricular ejection fraction, QRS interval, etc.) were not included in the statistical analysis because they were missing more than 60% of the data, and this would call for more in‐depth studies.

## 5 CONCLUSIONS

We have developed and internally validated a good discrimination decision tree model based on seven factors to predict the risk of IHCA, enabling patients to be readily stratified into three groups of high, intermediate, and low risk of CA. This simple prediction model may provide healthcare workers with a practical bedside tool, and will impact decision‐making for patients with ACS whose health is deteriorating.

## CONFLICT OF INTEREST

The authors declare no potential conflict of interest.

## AUTHORS' CONTRIBUTIONS

H.L. and Y.S.G. contributed in study design and definition of intellectual content. P.C.L., L.P.X., Y.C., D.L.Y. did the data acquisition, data analysis, and statistical analysis. T.T.W. contributed in the literature research, data acquisition and, manuscript drafting. T.T.W is the guarantor of integrity of the entire study and helped in data acquisition. H.L. designed the study concepts, manuscript review, and was also the guarantor of integrity of the entire study. All authors contributed to the approval of the final version.

## ETHICS STATEMENT

The Ethics Committee Board of Fujian Provincial Hospital approved this study and waived the requirement for written informed consent.
